# Characteristics of the synovial microenvironment and synovial mesenchymal stem cells with hip osteoarthritis of different bone morphologies

**DOI:** 10.1186/s13075-023-03252-y

**Published:** 2024-01-10

**Authors:** Yang Yang, Hideyuki Koga, Yusuke Nakagawa, Tomomasa Nakamura, Hiroki Katagiri, Ryohei Takada, Mai Katakura, Kunikazu Tsuji, Ichiro Sekiya, Kazumasa Miyatake

**Affiliations:** 1 Department of Joint Surgery and Sports Medicine, Tokyo Medical and Dental University, Tokyo, Japan; 2 Department of Cartilage Regeneration, Tokyo Medical and Dental University, Tokyo, Japan; 3 Department of Orthopaedic Surgery, Tokyo Medical and Dental University, Tokyo, Japan; 4 Center for Stem Cell and Regenerative Medicine, Tokyo Medical and Dental University, Tokyo, Japan; 5 Department of Orthopaedic Surgery, Dokkyo Medical University Saitama Medical Center, Saitama, Japan

**Keywords:** Hip osteoarthritis, Synovial mesenchymal stem cells, Bone morphology, RNA sequencing analysis, Cytokine, Osteophyte formation

## Abstract

**Background:**

Variations in bone morphology in patients with hip osteoarthritis (HOA) can be broadly categorized into three types: atrophic, normotrophic, and hypertrophic. Despite the investigations examining clinical elements, such as bone morphology, pain, and range of motion, our understanding of the pathogenesis of HOA remains limited. Previous studies have suggested that osteophytes typically originate at the interface of the joint cartilage, periosteum, and synovium, potentially implicating synovial mesenchymal stem cells (SMSCs) in the process. This study aimed to investigate the potential factors that drive the development of bone morphological features in HOA by investigating the characteristics of the synovium, differentiation potential of SMSCs, and composition of synovial fluid in different types of HOA.

**Methods:**

Synovial tissue and fluid were collected from 30 patients who underwent total hip arthroplasty (THA) with the variable bone morphology of HOA patients. RNA sequencing analysis and quantitative reverse transcription-polymerase chain reaction (RT-qPCR) were performed to analyse the genes in the normotrophic and hypertrophic synovial tissue. SMSCs were isolated and cultured from the normotrophic and hypertrophic synovial tissues of each hip joint in accordance with the variable bone morphology of HOA patients. Cell differentiation potential was compared using differentiation and colony-forming unit assays. Cytokine array was performed to analyse the protein expression in the synovial fluid.

**Results:**

In the RNA sequencing analysis, 103 differentially expressed genes (DEGs) were identified, predominantly related to the interleukin 17 (IL-17) signalling pathway. Using a protein–protein interaction (PPI) network, 20 hub genes were identified, including *MYC*, *CXCL8*, *ATF3*, *NR4A1*, *ZC3H12A*, *NR4A2*, *FOSB*, and *FOSL1*. Among these hub genes, four belonged to the *AP-1* family. There were no significant differences in the tri-lineage differentiation potential and colony-forming capacity of SMSCs. However, RT-qPCR revealed elevated *SOX9* expression levels in synovial tissues from the hypertrophic group. The cytokine array demonstrated significantly higher levels of CXCL8, MMP9, and VEGF in the synovial fluid of the hypertrophic group than in the normotrophic group, with *CXCL8* and *MMP9* being significantly expressed in the hypertrophic synovium.

**Conclusion:**

Upregulation of *AP-1* family genes in the synovium and increased concentrations of CXCL8, MMP9, and VEGF were detected in the synovial fluid of the hypertrophic group of HOA patients, potentially stimulating the differentiation of SMSCs towards the cartilage and thereby contributing to severe osteophyte formation.

**Supplementary Information:**

The online version contains supplementary material available at 10.1186/s13075-023-03252-y.

## Introduction

Hip osteoarthritis (HOA) is a degenerative joint disease characterized by the progressive degeneration of the joint cartilage, subchondral bone hardening, and osteophyte formation [1]. The diagnosis and classification of this disease significantly rely on the application of imaging technology. According to the Bombelli radiographic classification, HOA can be categorized into three subtypes: atrophic, normotrophic, and hypertrophic [2–4]. Notably, both normotrophic and hypertrophic HOA exhibit osteophyte formation. However, in hypertrophic HOA, enlargement of the femoral head and incidence of osteophyte formation are markedly higher than those in the normotrophic subtype [5].

Currently, the precise mechanism underlying osteophyte formation in arthritis remains unclear. It is widely accepted that osteophyte formation is primarily induced by inflammation that guides mesenchymal stem cells (MSCs) to the injury site [6]. In the damaged regions, these cells differentiate into chondrocytes, thus forming irregular cartilage tissue. Ultimately, ossification occurs within the cartilage, resulting in hardened osteophytes [7]. This process typically occurs at the junction of the joint cartilage, periosteum, and synovium, with synovial mesenchymal stem cells (SMSCs) potentially playing a role [8]. SMSCs possess unique differentiation and proliferative capabilities, and their differentiation type and capacity can vary under the regulation of various cell factors, making them a significant point of emphasis in musculoskeletal research [9].

This study aimed to investigate the potential factors that drive the development of bone morphological features in HOA. By comparing the characteristics of the synovium, differentiation potential of SMSCs, and composition of synovial fluid in normotrophic and hypertrophic HOA, we aimed to elucidate their pivotal roles in osteophyte formation. We hypothesized that the synovium, SMSCs, and synovial fluid in different types of HOA possess varying characteristics.

## Materials and methods

### Patient information

We evaluated patients undergoing total hip arthroplasty (THA) for HOA at Tokyo Medical and Dental University Hospital and collected patient samples. Patients with post-traumatic HOA, HOA following infection or osteonecrosis, and those with a history of osteotomy were excluded. Subsequently, patients were classified based on preoperative anteroposterior radiographs of both hips according to Bombelli classification [2]. Ultimately, we included normotrophic (osteophytes < grade 3) and hypertrophic (osteophytes ≥ grade 3 ± increased femoral head size) cases for comparison. Three orthopaedic surgeons independently evaluated the patients according to the Bombelli classification, requiring a consensus of at least 2. When there was a disagreement, the majority opinion was adopted. The demographic details of the patients, along with the classification outcomes, and the specific experiments conducted for each case are presented in Table 1. The study involved 30 participants (6 males and 24 females): 16 in the normotrophic group, 14 in the hypertrophic group, and none in the atrophic group. The mean age was 68.77 ± 8.14 years, with ages ranging from 50 to 88 years. There were no significant differences in terms of age (66.69 ± 8.07, 71.14 ± 7.83, *p* = 0.138) and body mass index (BMI; 25.49 ± 4.27, 24.35 ± 3.89, *p* = 0.759) across the 2 groups.

**Table 1:** Patient characteristics

No	Age, years	Sex	BMI	Side	Type	RNA-Seq	TLD	CFU	RT-qPCR	CA
1	58	F	26.16	Rt	N		✓		✓	
2	71	F	24.06	Rt	N	✓	✓			
3	66	F	34.52	Rt	N	✓	✓			
4	61	F	23.93	Lt	N		✓			
5	50	F	20.61	Lt	N	✓	✓	✓	✓	
6	59	F	34.48	Lt	N		✓	✓		
7	73	M	27.78	Rt	N		✓	✓		
8	66	F	23.11	Rt	N		✓	✓	✓	✓
9	72	F	23.99	Rt	N		✓	✓		
10	64	F	20.51	Rt	N		✓	✓	✓	✓
11	66	F	29.77	Rt	N		✓	✓	✓	
12	59	F	21.20	Rt	N		✓	✓		
13	82	M	23.77	Rt	N				✓	✓
14	72	M	23.74	Lt	N				✓	✓
15	76	M	24.30	Rt	N				✓	✓
16	72	F	25.97	Rt	N				✓	✓
17	66	F	30.35	Rt	H	✓	✓			
18	74	M	24.72	Rt	H		✓			
19	74	F	24.11	Rt	H	✓	✓		✓	
20	66	F	21.00	Lt	H		✓			
21	55	F	18.13	Rt	H	✓	✓	✓	✓	✓
22	75	F	25.54	Lt	H		✓	✓		
23	72	F	20.32	Rt	H		✓	✓	✓	
24	76	F	25.22	Lt	H		✓	✓		
25	70	F	21.23	Lt	H		✓	✓	✓	
26	80	F	25.46	Rt	H		✓	✓	✓	✓
27	67	F	20.55	Rt	H		✓	✓	✓	✓
28	65	M	29.84	Rt	H		✓	✓	✓	✓
29	88	F	30.40	Rt	H				✓	✓
30	68	F	23.97	Lt	H				✓	✓

*BMI* Body mass index (kg/m^2^), *CA* Cytokine array, *CFU* Colony-forming unit, *F* Female, *H* Hypertrophic, *Lt* Left, *M* Male, *N* Normotrophic, *RNA-Seq* RNA sequencing, *RT-qPCR* Quantitative reverse transcription-polymerase chain reaction, *Rt* Right, *TLD* Trilineage differentiation

### Patient-derived synovium and synovial fluid

Synovial tissues and fluids were harvested from the hip joints of patients with HOA during THA. Synovial tissue was collected from around the femoral neck. To ensure consistency in the sample collection, all procedures were conducted by the same surgical team. SMSCs were isolated from harvested synovial tissue following established protocols [10]. Briefly, synovial tissue was digested in a collagenase solution (Roche Diagnostics, Indianapolis, IN, USA) in α-Minimal Essential Medium (αMEM; Invitrogen, Waltham, MA, USA) at 37 °C for 3 h. Digested cells were filtered through a 70-μm cell strainer (Greiner Bio-One GmbH, Kremsmünster, Austria), and the remaining tissues were discarded. To obtain a sufficient number of cells, the nucleated cells were counted and plated at densities of 10^5^ cells in a 150-cm^2^ culture dish (Thermo Fisher Scientific Inc., Waltham, MA, USA) in complete culture medium: αMEM containing 10% foetal bovine serum (Invitrogen), 100 units/mL penicillin (Invitrogen), 100 mg/mL streptomycin (Invitrogen), and 250 ng/mL amphotericin B (Invitrogen). The cells were cultured at 37 °C in a humidified atmosphere of 5% CO_2_, with the remaining atmospheric composition mirroring normal air, which includes an oxygen concentration of approximately 21%, and prepared for subsequent experiments. The synovial fluids were centrifuged at 3000* g* to remove excess cells. For subsequent experimentations, the synovial fluids and remaining tissues were stored at − 80 °C.

### RNA extraction and RNA sequencing analysis

To investigate the gene expression differences in synovial tissues, we performed RNA sequencing analysis using samples from three donors. Total RNA, initially extracted from snap-frozen samples using TRIzol Reagent (Invitrogen), was further purified using a NucleoSpin RNA kit (Macherey–Nagel, Dueren, Germany). RNA quantification was achieved using a NanoDrop 1000 spectrophotometer (Thermo Fisher Scientific Inc.). Absorbance ratios of approximately 2.0 for 260/280 and 1.8–2.2 for 260/230 were indicative of pure RNA, free from protein and organic compound contamination. After purification, the samples were shipped to a commercial service provider for library preparation. The RNA integrity number (RIN) of extracted RNA was assessed using an Agilent 2100 Bioanalyzer (Agilent Technologies, Santa Clara, CA, USA), and samples with a RIN value of 7.0 or higher were considered suitable for subsequent RNA sequencing. Illumina NovaSeq 6000 platform (Illumina, San Diego, CA, USA) was used for sequencing. After sequencing, the service provider performed an initial quality assessment using FastQC (Illumina), adaptor trimming using Trimmomatic (http://usadellab.org), and alignment with the reference genome using hierarchical indexing for spliced alignment of transcripts (HISAT2). Finally, gene read counts were determined using the featureCounts function in the subread package.

### Screening for differentially expressed genes (DEGs)

We conducted a differential analysis based on gene read counts and identified DEGs using the R package “DEseq2”. The criteria for DEG screening were set as an adjusted *p*-value < 0.05 and |log2 fold change|> 1.

### Functional enrichment analysis and protein–protein interaction (PPI) network analysis of DEGs

For functional enrichment analysis of DEGs and subsequent PPI network analysis, Gene Ontology (GO) and Kyoto Encyclopedia of Genes and Genomes (KEGG) pathway enrichment analyses were performed using the “clusterProfiler” R package, with cutoff values for *p* and *q* set at 0.05. The results from the enrichment analysis were visualized using “ggpubr”, “ggplot2”, and “Goplot” R packages. To construct PPI networks, we used the STRING database (https://string-db.org/) and exported the resulting network diagrams. These results were imported into the Cytoscape software (version 3.10.0), and key nodes were selected for visualization of the molecular interaction network. Finally, we used the CytoHubba plugin to identify key genes based on the constructed PPI network.

### Differentiation assays

#### Adipogenesis

The adipogenic potential of SMSCs was evaluated by established methods using samples from 12 donors [10]. Briefly, 100 passage 1 cells were cultured in 60-cm^2^ dishes with complete αMEM medium for 14 days. The medium was then switched to adipogenic medium consisting of αMEM supplemented with 10% foetal bovine serum (Invitrogen), 10^−7^ M dexamethasone (Sigma-Aldrich, St. Louis, MO, USA), 0.5 mM isobutyl-1-methylxanthine (Sigma-Aldrich), and 100 mM indomethacin (FUJIFILM Wako Chemicals. Richmond, VA, USA), for an additional 21 days. Following fixation in 10% formalin (Wako), the cells were stained with oil red O (Sigma-Aldrich), and colonies were counted via the National Institutes of Health (NIH) ImageJ software. The dishes were counterstained with crystal violet for total colony visualization. The rate of oil red O-positive colonies was determined by dividing the number of oil red O-positive colonies by the total number of colonies, and colonies < 2 mm in diameter were discounted.

#### Calcification

The calcification potential of SMSCs was evaluated by established methods using samples from 12 donors [10]. Briefly, 100 passage 1 cells were cultured in 60-cm^2^ dishes with complete αMEM medium for 14 days. The medium was then switched to an osteogenic differentiation medium consisting of αMEM supplemented with 10% foetal bovine serum (Invitrogen), 10^−9^ M dexamethasone (Sigma-Aldrich), 10 mM b-glycerol phosphate (Wako), and 50 mg/mL ascorbate-2-phosphate (Sigma-Aldrich) for an additional 21 days. Following fixation in 10% formalin (Wako), the cells were stained with alizarin red S (Sigma-Aldrich), and colonies were counted using the NIH ImageJ software. The dishes were then counterstained with crystal violet for total colony visualization. The rate of alizarin red S-positive colonies was determined by dividing the alizarin red S-positive colonies by the total number of colonies. Colonies < 2 mm in diameter were discounted.

#### Chondrogenesis

The chondrogenic potential of SMSCs was evaluated by established methods using samples from 12 donors [10]. A total of 250,000 passage 1 cells were placed in a 15-mL polypropylene tube (Falcon®; Corning Inc., Corning, NY, USA) and centrifuged at 1500 rpm for 5 min. Subsequently, the cells were cultured in chondrogenic medium: high-glucose Dulbecco’s modified Eagle’s medium (Invitrogen) supplemented with 5000 ng/mL BMP-2 (Sigma-Aldrich), 10 ng/mL TGF-b3 (R&D Systems, Minneapolis, MN, USA), 10^−7^ M dexamethasone (Sigma-Aldrich), 50 mg/mL ascorbate2-phosphate (Sigma-Aldrich), 40 mg/mL proline (Sigma-Aldrich), 100 mg/mL pyruvate (Sigma-Aldrich), and 5 mg/mL ITS 1 Premix (Becton, Dickinson and Company, Franklin Lakes, NJ, USA), which was replaced every 3–4 days for 21 days. The chondrogenic pellets were fixed with 4% PFA and photographed, and the Feret diameters of the pellets were measured using the NIH ImageJ software to compare their sizes.

### Colony-forming unit assay (CFU)

The colony-forming potential of SMSCs was evaluated by established methods using samples from 8 donors [10]. Briefly, 100 passage 1 cells were cultured in 60-cm^2^ dishes with complete αMEM medium for 14 days. Following fixation in 10% formalin, the cells were stained with crystal violet, and colonies were counted using the NIH ImageJ software. Colonies < 2 mm in diameter were discounted.

### Cytokine array

For the cytokine array, we analysed the cytokine profiles of samples provided by six donors using the Proteome Profiler Human XL Cytokine Array (R&D Systems, Minneapolis, MN, USA). Briefly, the synovial fluid was combined with a biotinylated detection antibody cocktail and incubated with a nitrocellulose membrane. After washing, the membranes were exposed to streptavidin–horseradish peroxidase. Cytokines were detected using the ChemiDoc XRS + system (Bio-Rad, Hercules, CA, USA). The signal density of each imprint was quantified using dot plot analysis on the NIH ImageJ software, subtracting the background intensity, and finally calculating the values as a percentage of the reference spot intensity. If the final percentage calculated value of both groups was less than 0.05 at the same time, the cytokine was regarded as having low secretion, and its *p*-value was not calculated.

### Quantitative reverse transcription-polymerase chain reaction (RT-qPCR)

Total RNA was extracted from the synovial tissue of nine donors per group using TRIzol Reagent and subsequently purified using a NucleoSpin RNA kit. RNA quantification was achieved using a NanoDrop 1000 spectrophotometer (Thermo Fisher Scientific Inc.). Absorbance ratios of approximately 2.0 for 260/280 and 1.8–2.2 for 260/230 were indicative of pure RNA, which was considered suitable for subsequent experiments. Complementary cDNA was synthesized from purified RNA using a reverse transcription kit (Toyobo, Osaka, Japan). RT-qPCR was performed on a LightCycler 480 (Roche, Basel, Switzerland) using TaqMan Gene Expression Assays (Applied Biosystems, Thermo Fisher Scientific, Waltham, MA, USA). Relative gene expression levels were normalized to actin beta (ACTB) and calculated using the 2 − ΔΔCt method. Duplicate runs were performed for each sample. The reagents used in the TaqMan assays were ACTB (Hs01060665_g1), ANGPTL4 (Hs01101127_m1), BMP2 (Hs00154192_m1), COL1A1 (Hs00164004_m1), CXCL2 (Hs00601975_m1), CXCL3 (Hs00171061_m1), CXCL8 (Hs00174103_m1), FOSL1 (Hs00759776_s1), HAS1 (Hs00758053_m1), MMP9 (Hs00957562_m1), RUNX2 (Hs00231692_m1), SOX9 (Hs00165814_m1), and VEGFA (Hs00900055_m1).

### Statistical analysis

Statistical analyses were performed using the R software (version 4.1.2; R Foundation for Statistical Computing, Vienna, Austria) or GraphPad Prism (version 7.0; GraphPad Software, Inc., San Diego, CA, USA). The Shapiro–Wilk test was used to assess whether the data were normally distributed. Unpaired Student’s *t* test and Welch’s *t* test were used for normally distributed values. The Mann–Whitney *U* test was used for values with non-normal distributions. In correlation analysis, the correlation coefficient *r* represents varying degrees of linear relationship: a value between 0 and 0.3 (or − 0.3 and 0) signifies a weak positive (negative) linear correlation, a value between 0.3 and 0.7 (or − 0.7 and − 0.3) signifies a moderate positive (negative) linear correlation, and a value between 0.7 and 1.0 (or − 1.0 and − 0.7) indicates a strong positive (negative) linear correlation [11]. Statistical significance was set at *p* < 0.05.

## Results

### Differential gene expression analysis

**Fig. 1:**
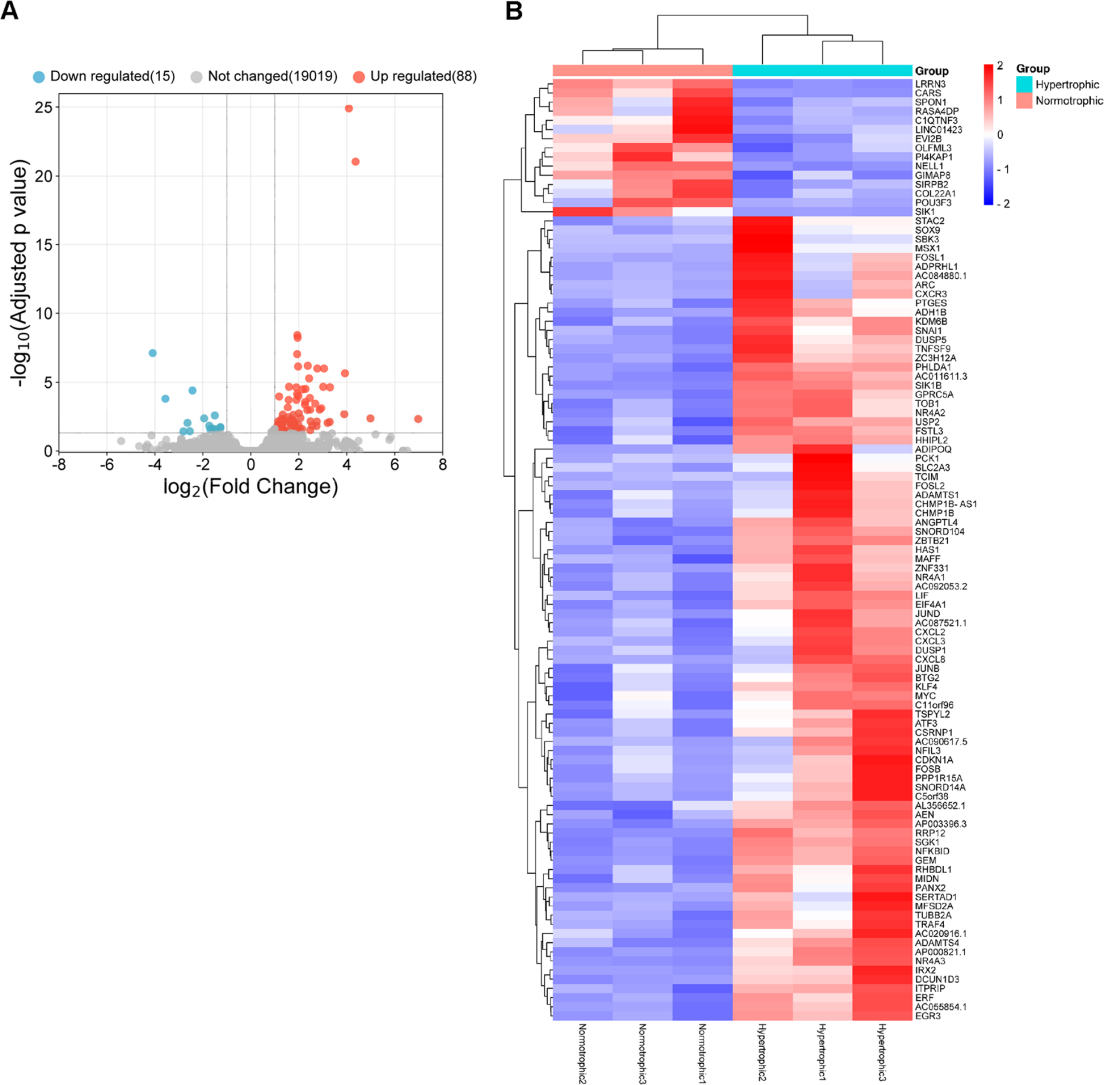
RNA sequencing of synovial tissue from normotrophic and hypertrophic patients. **A** Volcano plot of 103 differentially expressed genes (DEGs). **B** Heatmap of DEGs in synovial tissue

RStudio (RStudio, Inc., Boston, MA, USA) was used to analyse the DEGs between synovial tissues from the hypertrophic and normotrophic groups. DESeq2 was used for comparison and analysis, with the normotrophic group serving as a control. Genes with adjusted *p*-value < 0.05 and |log2 fold change|> 1 were considered DEGs. A total of 103 DEGs were identified, including 88 upregulated and 15 downregulated DEGs. A volcano plot was generated to verify these results (Fig. 1A). The raw read counts of these DEGs were converted to transcripts per million (TPM) values to generate a heat map of gene expression (Fig. 1B).

### GO and KEGG enrichment analysis of DEGs

**Fig. 2:**
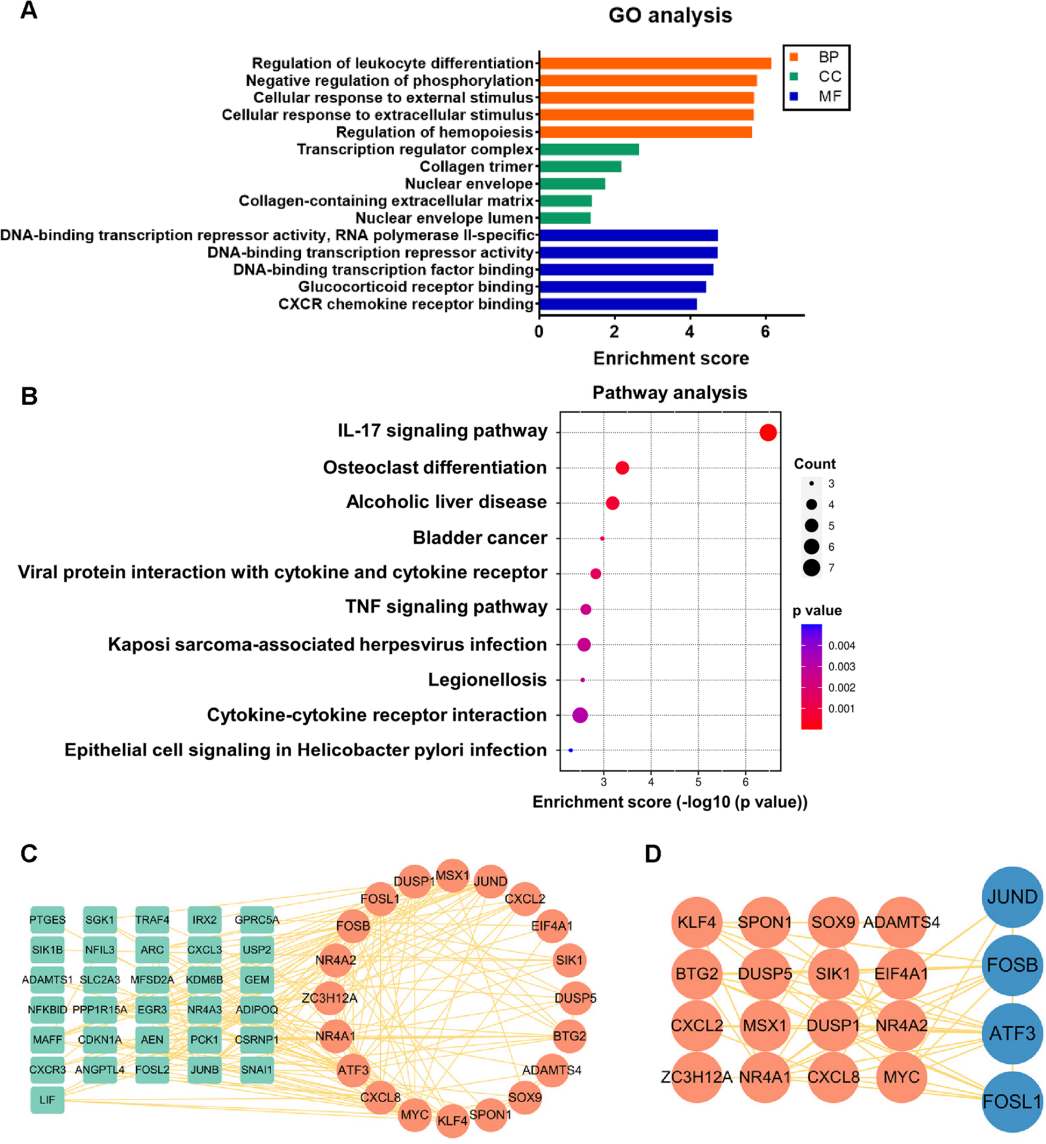
Enrichment analysis and protein–protein interaction (PPI) analysis. **A** Gene Ontology (GO) enrichment analysis bar plot of 103 DEGs. **B** Kyoto Encyclopedia of Genes and Genomes (KEGG) pathway dot plot analysis of 103 DEGs. **C** PPI analysis with betweenness algorithm. Red nodes: top 20 hub genes. Green nodes: second-level key genes. **D** AP-1 family members are marked in blue in the top 20 hub genes

To investigate the potential biological functions and signalling pathways of the DEGs, GO and KEGG enrichment analyses were performed using the R package “clusterProfiler”. GO analysis showed that the DEGs were mainly enriched in biological processes (BP), such as regulation of leukocyte differentiation, negative regulation of phosphorylation, and transcription regulator complex; cellular components (CC), such as cellular response to external stimulus, collagen trimer, and nuclear envelope; and molecular functions (MF), such as DNA-binding transcription repressor activity, RNA polymerase II-specific, DNA-binding transcription repressor activity, and DNA-binding transcription factor binding (Fig. 2A). KEGG pathway enrichment analysis revealed that these 103 DEGs were primarily related to the interleukin 17 (IL-17) signalling pathway and osteoclast differentiation (Fig. 2B).

### PPI network analysis and key gene selection

We used the STRING database to analyse the PPI network of these 103 DEGs. Based on the STRING database, a Cytoscape network visualization map was obtained (Fig. 2C), which included 51 nodes and 336 edges. Using the CytoHubba plugin of the Cytoscape software, we arranged these 51 nodes using the betweenness algorithm and marked 20 hub genes, including *MYC*, *CXCL8*, *ATF3*, *NR4A1*, *ZC3H12A*, *NR4A2*, *FOSB*, and *FOSL1*. Among these hub genes, four genes belonging to the *AP-1* family were identified (Fig. 2D).

### Analysis of gene expression in synovial tissue

**Fig. 3:**
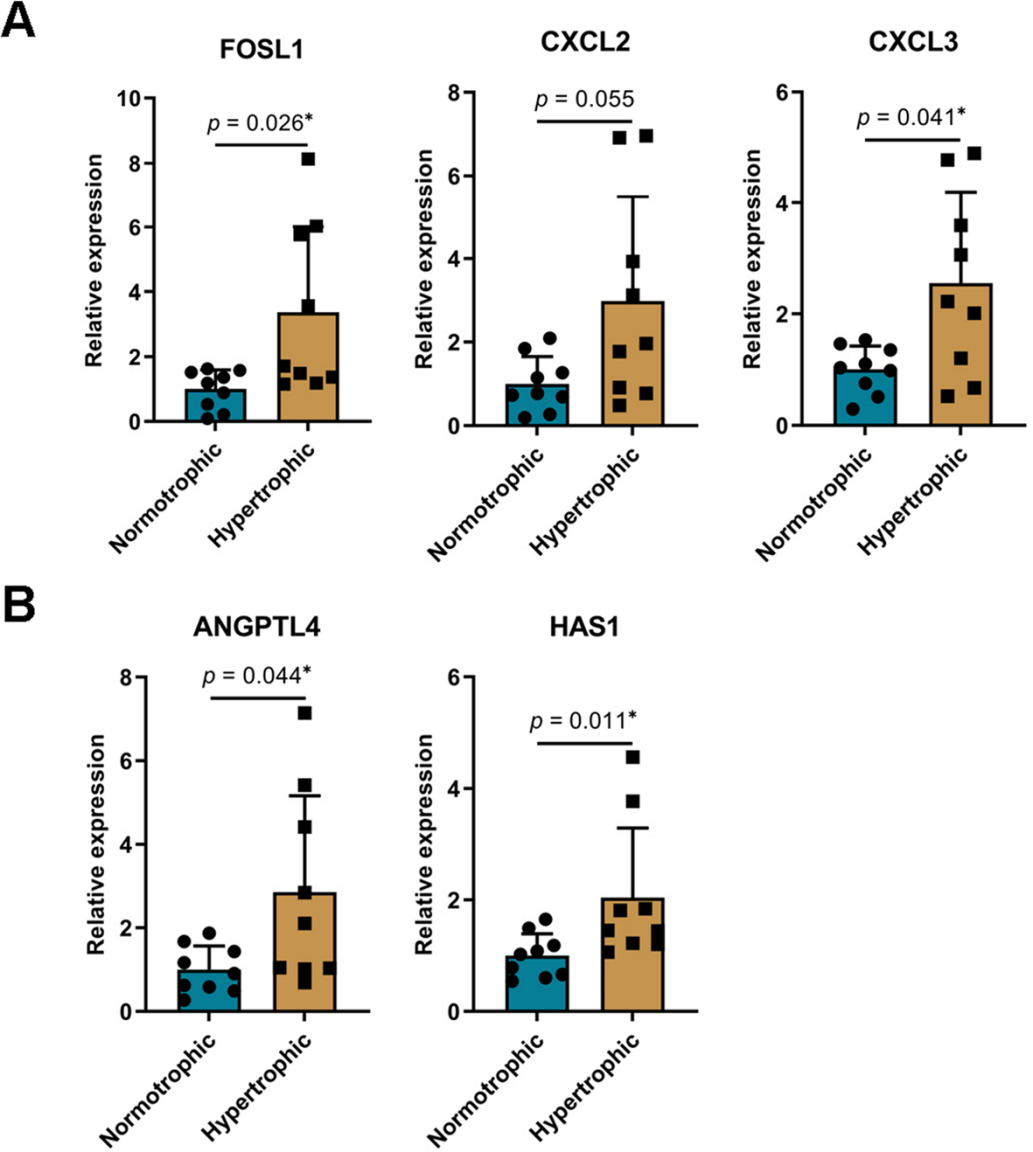
Analysis of gene expression in synovial tissue. **A** Relative expression of *AP-1*-related genes in synovium. **B** Relative expression of *ANGPTL4* and *HAS1* in synovium. The data are expressed as mean ± SD, ^∗^*p* < 0.05

Guided by the outcomes of the PPI network screening, the expression of additional crucial genes was further investigated. These include *FOSL1*, *CXCL2*, and *CXCL3*, which are related to *AP-1. ANGPTL4* implicated in the chondrogenic differentiation of MSCs, whereas *HAS1* has been associated with osteophyte formation and arthritis (Fig. 3). Our findings revealed that the hypertrophic group displayed significantly higher expression levels of *FOSL1*, *CXCL3*, *ANGPTL4*, and *HAS1* in the synovial tissues than the normotrophic group.

### Adipogenesis

**Fig. 4:**
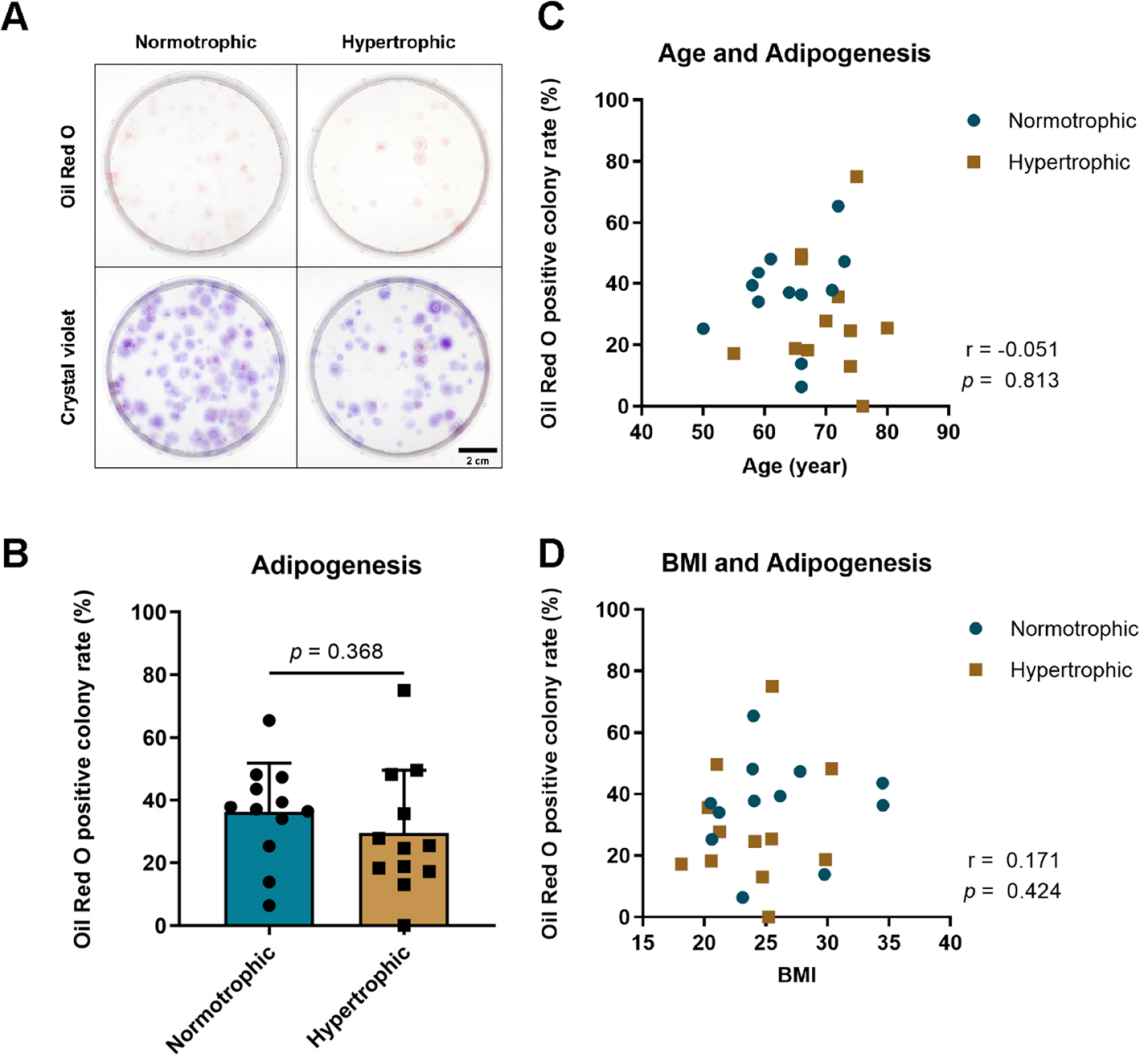
Adipogenesis of synovial mesenchymal stem cells (SMSCs) in normotrophic and hypertrophic groups. **A** Oil red O and crystal violet staining in cells. **B** Comparison of oil red O-positive colony rates. **C** Correlation of adipogenesis with age. **D** Correlation of adipogenesis with body mass index (BMI). The data are expressed as mean ± SD

All samples produced adipocyte colonies, which were subsequently stained with oil red O (Fig. 4). No significant differences were observed in the ratios of oil red O-positive colonies. No significant correlation was found between adipogenesis and age or BMI.

### Calcification

**Fig. 5:**
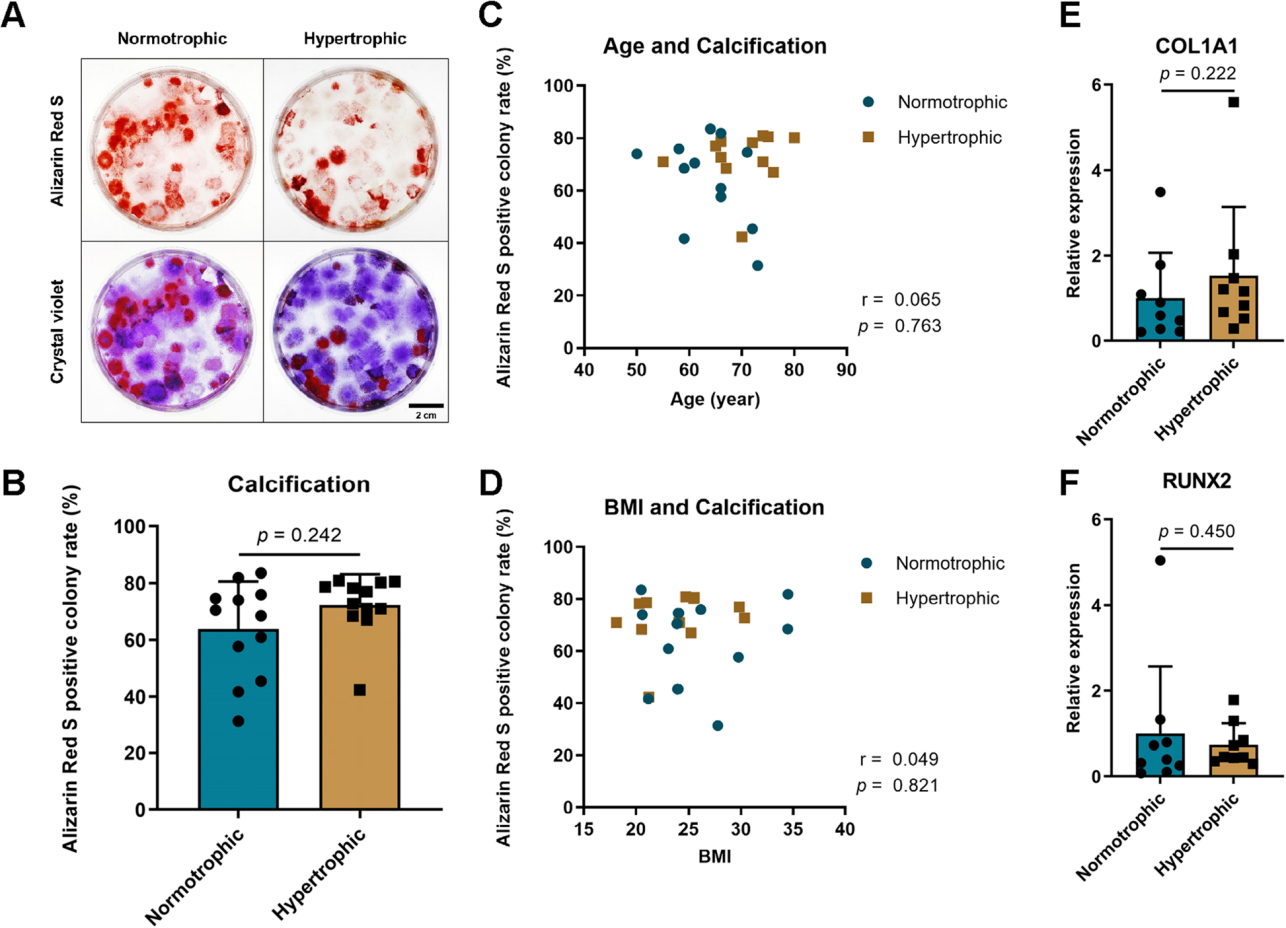
Calcification of synovial mesenchymal stem cells (SMSCs) in normotrophic and hypertrophic groups. **A** Alizarin red S and crystal violet staining in cells. **B** Comparison of alizarin red S-positive colony rates. **C** Correlation of calcification with age. **D** Correlation of calcification with body mass index (BMI). **E** Relative expression of *COL1A1* in synovium. **F** Relative expression of *RUNX2* in synovium. The data are expressed as mean ± SD

All samples produced colonies of calcification that were stained with Alizarin Red S (Fig. 5). No significant differences were observed in the ratios of Alizarin Red S-positive colonies. Correlation analysis was also performed to test the link between osteogenesis and factors such as age and BMI; no significant differences were found. Additionally, RT-qPCR analysis performed on synovial tissues from nine donors to evaluate osteogenic gene expression revealed that the levels of *COL1a1* and *RUNX2* in the synovial tissues of the two groups were not significantly different.

### Chondrogenesis

**Fig. 6:**
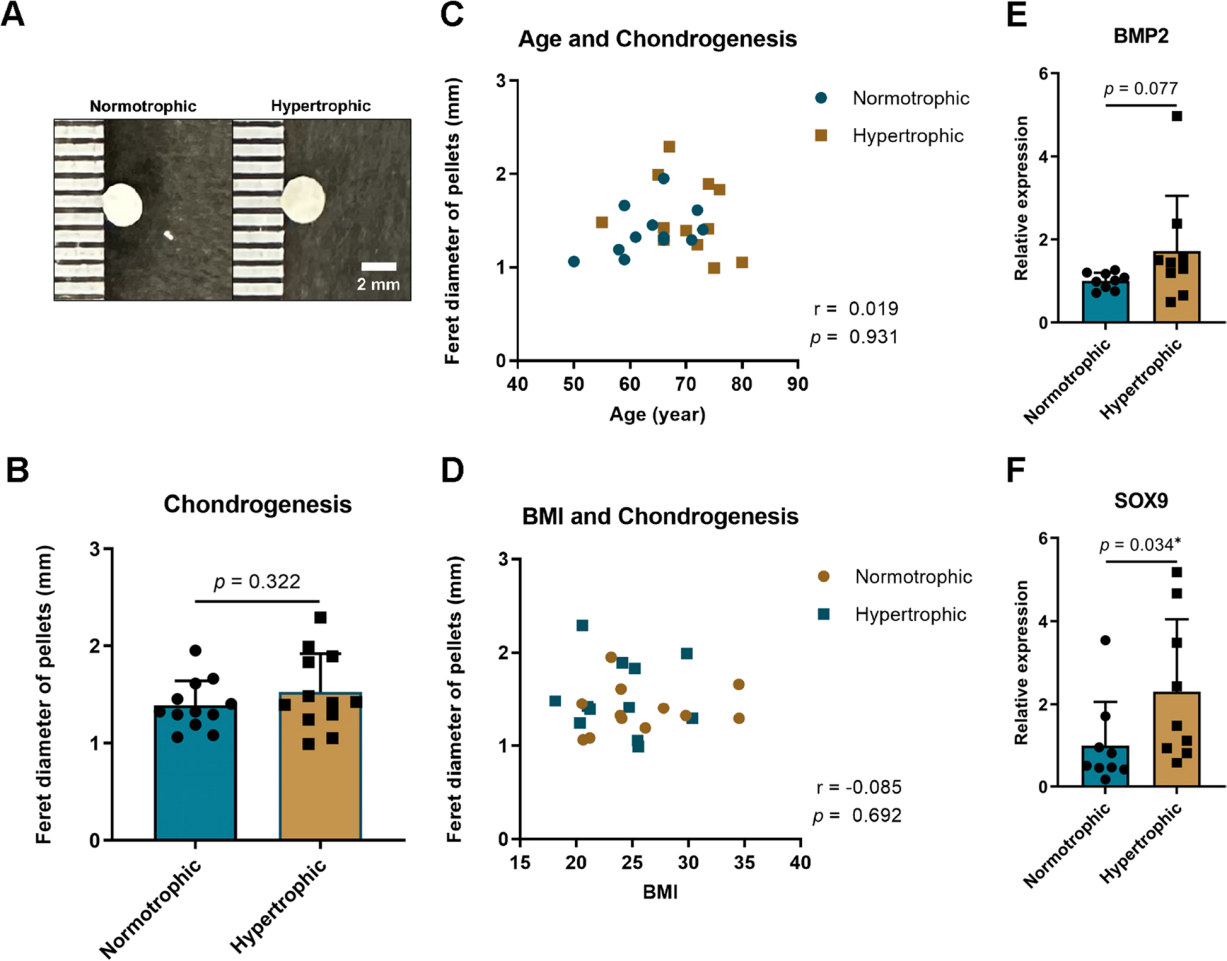
Chondrogenesis of synovial mesenchymal stem cells (SMSCs) in normotrophic and hypertrophic groups. **A** Macroscopic view of chondrogenic pellets. **B** Comparison of pellet diameters between groups. **C** Correlation of chondrogenesis with age. **D** Correlation of chondrogenesis with body mass index (BMI). **E** Relative expression of *BMP2* in synovium. **F** Relative expression of *SOX9* in synovium. The data are expressed as mean ± SD, ^∗^*p *< 0.05

The Feret diameter was used to compare the chondrogenic pellets (Fig. 6), and no significant differences were observed. A correlation analysis was also conducted to investigate the association between chondrogenesis and age or BMI, and no significant differences were identified. RT-qPCR revealed that the expression of *SOX9* in the synovial tissues of the hypertrophic group was higher than that in the normotrophic group. Although the expression of *BMP2* showed an increasing trend, it did not reach statistical significance. These results suggest that while there may not be a significant difference in the chondrogenic capability of SMSCs between the two groups, cartilage generation may occur in the synovial tissues of the hypertrophic group.

### Colony-forming unit assay

**Fig. 7:**
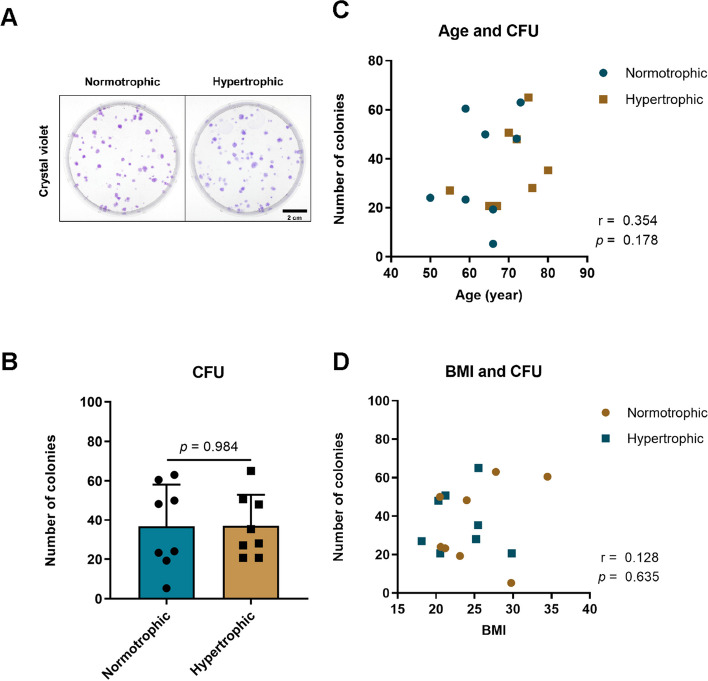
Colony-forming unit (CFU) of synovial mesenchymal stem cells (SMSCs) in normotrophic and hypertrophic groups. **A** Crystal violet staining in cells. **B** Comparison of number of colonies. **C** Correlation of CFU with age. **D** Correlation of CFU with body mass index (BMI). The data are expressed as mean ± SD

All samples produced SMSC colonies that were subsequently stained with crystal violet (Fig. 7). No significant differences were observed in the number of SMSC colonies. Furthermore, correlation analysis was performed to examine the association between CFU and variables such as age and BMI; however, no significant differences were found.

### Cytokine array analysis of synovial fluid

**Fig. 8:**
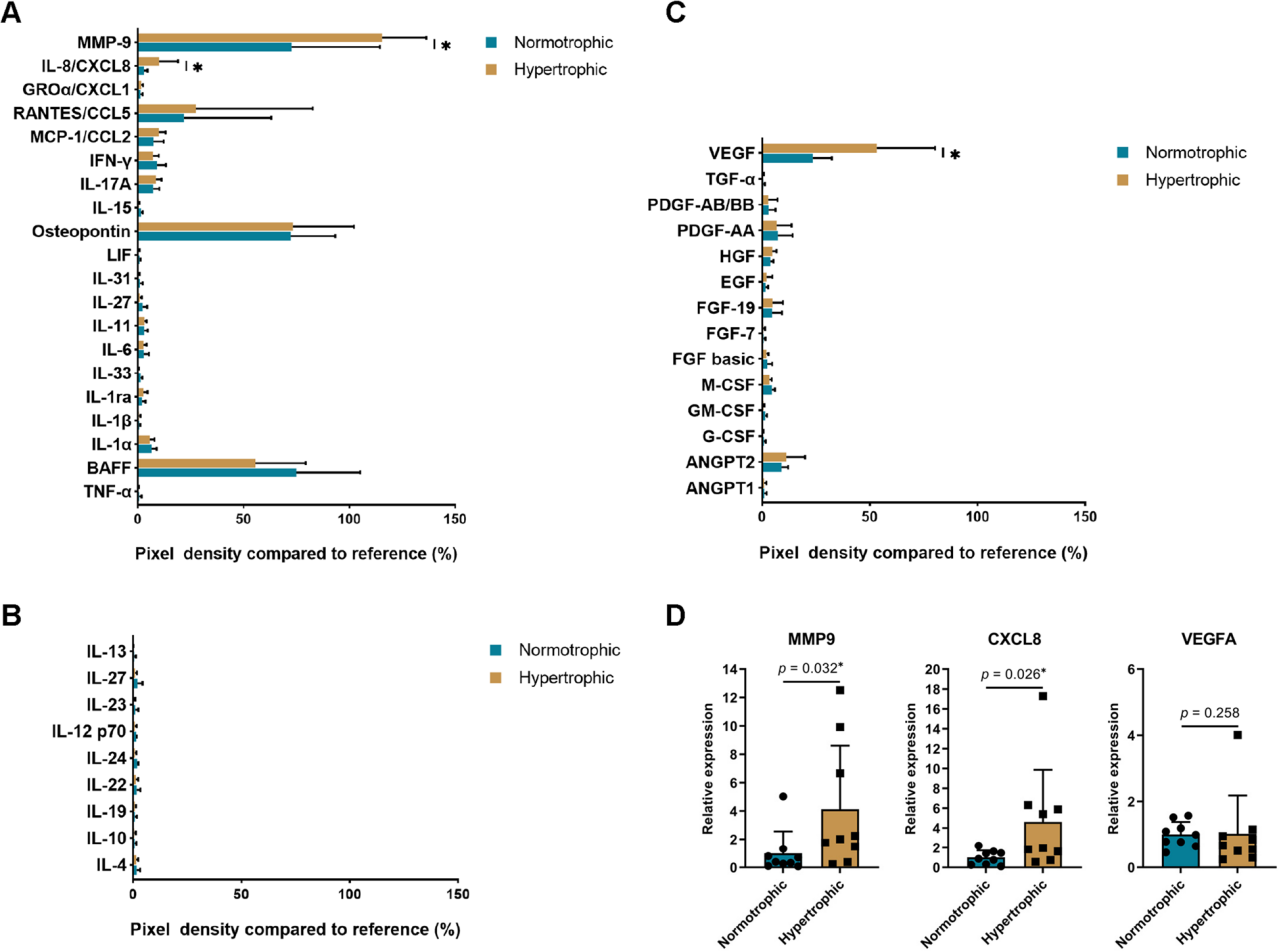
Cytokine expression of synovial fluid in normotrophic and hypertrophic patients. **A** Cytokines involved in pro-inflammatory responses in the synovial fluid. **B** Cytokines involved in anti-inflammatory responses in the synovial fluid. **C** Growth factors in the synovial fluid. **D** Relative expression of differentially expressed cytokines in the synovium. The data are expressed as mean ± SD, *p < 0.05

Cytokine array analysis was performed on the synovial fluid to analyse the protein components of the synovial fluid. The main cytokines were categorized into three groups: cytokines involved in pro-inflammatory responses, those involved in anti-inflammatory responses, and growth factors (Fig. 8A–C). The results showed that in the hypertrophic group, the expression levels of the pro-inflammatory factors, CXCL8 and MMP9, were significantly higher than those in the normotrophic group. Both synovial fluid samples showed the presence of interleukin-17A (IL-17A). However, IL-17A levels were not significant between the two groups. The levels of all cytokines involved in anti-inflammatory responses were extremely low. We also observed that the levels of the growth factor, VEGF, in the synovial fluid of the hypertrophic group were significantly higher than those in the normotrophic group. RT-qPCR analysis of synovial tissues from nine donors showed that the expression of *CXCL8* and *MMP9* in the hypertrophic group’s synovial tissues was significantly higher than that in the normotrophic group’s synovial tissues, whereas the level of *VEGFA* showed no significant difference (Fig. 8D). This suggests that CXCL8 and MMP9 found in the synovial fluid may be partially secreted from the synovial tissue.

## Discussion

Our study revealed that morphological alterations and osteophyte development in HOA are closely associated with the upregulation of key genes, such as the *AP-1* family, in the synovial fluid, along with increased concentrations of CXCL8, MMP9, and VEGF. These molecular changes were more pronounced in the hypertrophic group, suggesting their potential role in severe osteophyte formation. Morphological alterations and osteophyte development, viewed as potential indicators of HOA severity, are believed to be correlated with joint pain, range of motion, and the impact on activities of daily living [12, 13].

KEGG enrichment analysis suggested that the IL-17 signalling pathway may be implicated in HOA inflammation [14, 15]. By constructing a PPI network, we identified four hub DEGs that were screened as members of the *AP-1* family [16]. These genes not only participate in the IL-17 signalling pathway but also influence downstream cytokines and chemokines [17, 18]. Based on the genomic results, we measured the expression levels of genes in the synovium that might be involved in the IL-17 signalling pathway. The *AP-1* family gene *FOSL1* and its downstream pathway members, *CXCL3* and *CXCL8*, were significantly expressed in the synovium of the hypertrophic group.

In patients with late-stage HOA, no significant difference was observed in the chondrogenic differentiation capacity of SMSCs between the groups in our in vitro experiments. *BMP2* is closely associated with the chondrogenic differentiation of MSCs in synovial tissues [19, 20]. Our results showed an upward trend in *BMP2* expression in the hypertrophic group, without reaching statistical significance, compared to the normotrophic group. However, the expression of *SOX9*, a key gene involved in cartilage differentiation, was significantly increased in the hypertrophic group. This finding suggests that cartilage differentiation occurs in the synovium under these pathological conditions [21, 22]. Additionally, our in vitro investigations showed no significant differences in either tri-lineage differentiation or colony-forming capacity of SMSCs between the different groups. No correlation was established between these SMSC properties and patient-specific factors such as age or BMI. These observations are consistent with the outcomes from previous studies conducted on both rats and humans, which reported that the growth and differentiation capacity of MSCs in vitro are independent of donor age and weight [23, 24].

Moreover, through a cytokine array analysis of synovial fluid samples from both groups, IL-17A was detectable in the synovial fluid, as predicted by the genomic data. However, no significant difference in the IL-17A levels was observed between the two groups. We hypothesized that in the late stages of HOA, cytokines from the IL-17 family are indeed involved in joint inflammation, but the family member, IL-17A, might not be the key cytokine leading to distinct outcomes. Previous studies indicate that interleukin-1 beta (IL-1β) and tumour necrosis factor alpha (TNFα) are instrumental in the progression of osteoarthritis [25, 26]. Interestingly, in our study, these two pro-inflammatory factors were present at very low levels in the synovial fluid and were almost negligible. This is in line with other studies, which show a decline in the levels of IL-1β and TNFα in the late stages of osteoarthritis compared to earlier stages of the disease [27, 28]. The reason for this dramatic decline in metabolic activity in the late stages of the disease remains unclear; however, it could, at least in part, be attributed to cell death.

Cytokine array analysis revealed significantly higher levels of CXCL8, MMP9, and VEGF in the synovial fluid of the hypertrophic group than those in the normotrophic group. RT-qPCR results showed a significantly higher expression of *CXCL8* and *MMP9* in the hypertrophic synovium, whereas *VEGFA* expression remained unchanged, indicating that the synovium was a probable source of CXCL8 and MMP9 in the synovial fluid. Previous studies have shown that the AP-1 binding site at − 73 bp is a key regulator of *MMP9* transcription [29, 30]. This is consistent with our genomic analysis and RT-qPCR results, indicating that the expression of *AP-1* family genes in synovial tissue was higher in the hypertrophic group than in the normotrophic group. Increased CXCL8 levels correspond to heightened inflammation, which could enhance the proliferative capacity of SMSCs [31, 32]. Notably, VEGF can directly stimulate MSCs, enhancing their osteogenic and chondrogenic differentiation abilities, and thereby causing osteophyte formation and enlargement [33–35]. Based on our findings as well as existing research, VEGF inhibition is a potential therapeutic approach for knee osteoarthritis. Specifically, bevacizumab, a humanized monoclonal antibody against VEGF, has attracted attention because of its potential to alter the disease course by modulating these angiogenic pathways [36].

Furthermore, studies have shown that *HAS1* and *ANGPTL4* expression are closely associated with osteophyte formation and cartilage differentiation. *HAS1* is significantly expressed in osteophyte cartilage, and *ANGPTL4* is one of the most upregulated genes encoding secreted factors in the transforming growth factor beta (TGF-β)-induced cartilage formation process [37, 38]. In our transcriptome analysis, both genes were found to be differentially expressed between the two synovial groups. RT-qPCR results also showed increased expression of *HAS1* and *ANGPTL4* in the synovium of the hypertrophic group, suggesting the occurrence of cartilage differentiation and a potential osteophyte increase in the pathological process of the hypertrophic synovium.

Hence, we propose that within this microenvironment, SMSCs situated in the synovium at the interface between the articular cartilage and synovium may undergo chondrogenic differentiation. This can result in exacerbated osteophyte formation and, ultimately, distinct bone morphological alterations associated with HOA.

The limitations of the present study are as follows. First, it is difficult to describe the potential of the SMSCs in vivo from our in vitro study, and there was a lack of multiple validating experiments. Second, the patients in our cohort varied widely in terms of demographics, and the sample size of our study was limited. Third, not all experiments were conducted for each patient. Fourth, we did not investigate other joint components that might influence the progression of HOA, such as cartilage and bone marrow. Fifth, we did not compare our findings with those from healthy individuals or with other types of HOA, such as the atrophic type, which restricts the scope of our conclusions. Future studies should focus on detailed mechanistic research to enhance the understanding of the underlying processes.

## Conclusions

In conclusion, our observations indicate the upregulation of *AP-1* family genes in the synovium and increased concentrations of CXCL8, MMP9, and VEGF in the synovial fluid of the hypertrophic group among patients with HOA. Enhanced levels of these markers could potentially stimulate the chondrogenic differentiation of synovium-residing MSCs, thereby exacerbating osteophyte severity.

### Supplementary Information

**Additional file 1: Supplementary Figure S1. **Patient information.

## Data Availability

The datasets used and/or analysed during the current study are available from the corresponding author upon reasonable request.

## Abbreviations

ACTB: Actin beta
αMEM: α-Minimal Essential Medium
ANGPTL4: Angiopoietin-like 4
AP-1: Activator protein 1
BMI: Body mass index
BMP2: Bone morphogenetic protein 2
CFU: Colony-forming unit
COL1A1: Collagen type I alpha 1 chain
CXCL2: C-X-C motif chemokine ligand 2
CXCL3: C-X-C motif chemokine ligand 3
CXCL8: C-X-C motif chemokine ligand 8
DEGs: Differentially expressed genes
FOSL1: FOS-like antigen 1
GO: Gene Ontology
HAS1: Hyaluronan synthase 1
HOA: Hip osteoarthritis
IL-1β: Interleukin-1 beta
IL-17: Interleukin 17
IL-17A: Interleukin-17A
KEGG: Kyoto Encyclopedia of Genes and Genomes
MMP9: Matrix metallopeptidase 9
MSCs: Mesenchymal stem cells
PPI: Protein-protein interaction
RT-qPCR: Quantitative reverse transcription-polymerase chain reaction
RUNX2: Runt-related transcription factor 2
SMSCs: Synovial mesenchymal stem cells
SOX9: SRY-box transcription factor 9
THA: Total hip arthroplasty
TNFα: Tumour necrosis factor alpha
VEGFA: Vascular endothelial growth factor A
