# Supplementary material for: Characteristics of the synovial microenvironment and synovial mesenchymal stem cells with hip osteoarthritis of different bone morphologies
Source: Arthritis Res Ther. 2024 Jan 10;26:17. doi: 10.1186/s13075-023-03252-y (PMC10777653; doi:10.1186/s13075-023-03252-y)
Supplement: Supplementary file 1 — Additional file 1: Supplementary Figure S1. Patient information. [file 13075_2023_3252_MOESM1_ESM.pptx]

## Slide 1
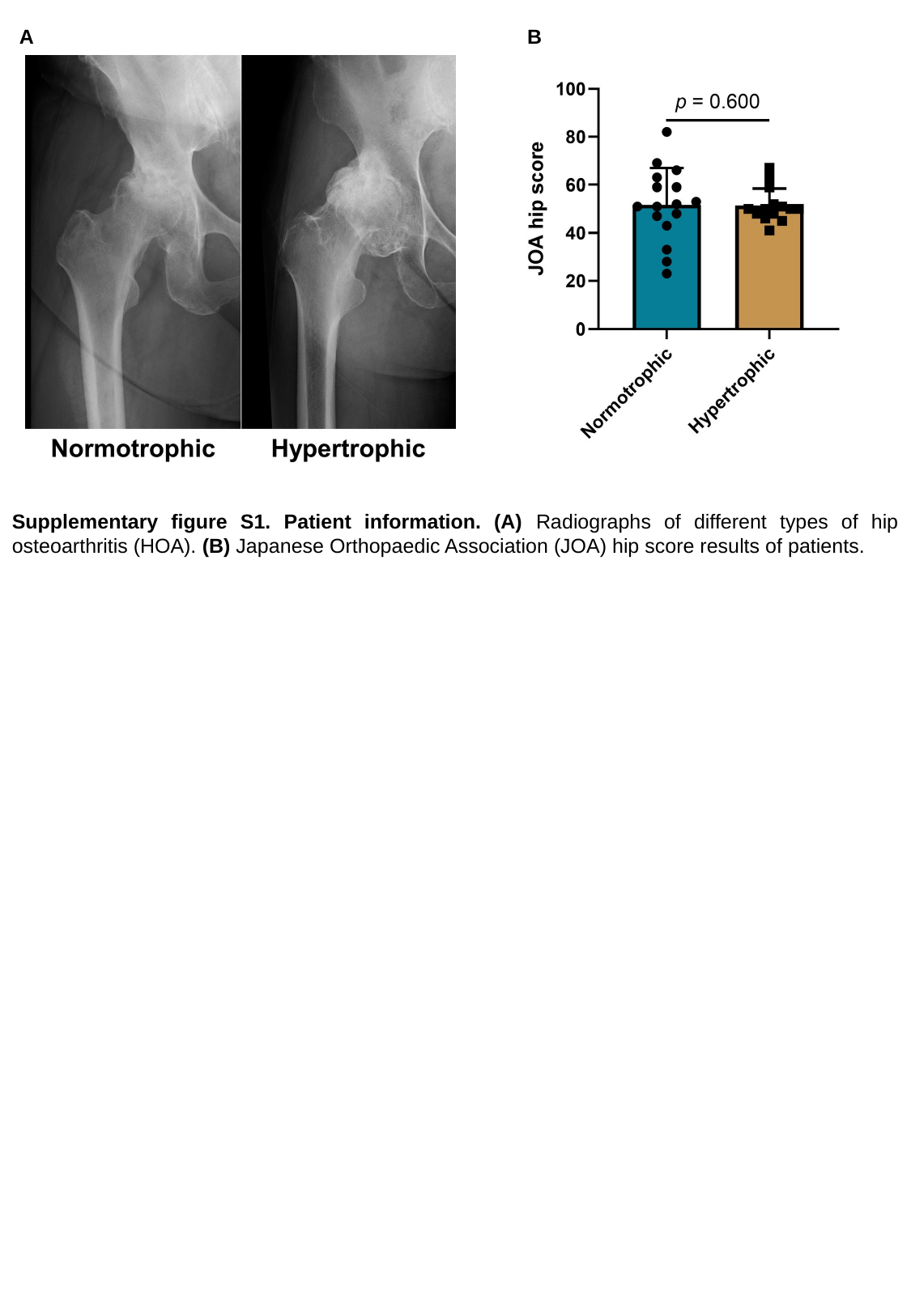

A
B
Supplementary figure S1. Patient information. (A) Radiographs of different types of hip osteoarthritis (HOA). (B) Japanese Orthopaedic Association (JOA) hip score results of patients.
